# MexEF-OprN Efflux Pump Exports the *Pseudomonas* Quinolone Signal (PQS) Precursor HHQ (4-hydroxy-2-heptylquinoline)

**DOI:** 10.1371/journal.pone.0024310

**Published:** 2011-09-21

**Authors:** Martin G. Lamarche, Eric Déziel

**Affiliations:** INRS-Institut Armand-Frappier, Laval, Québec, Canada; National Institutes of Health, United States of America

## Abstract

Bacterial cells have evolved the capacity to communicate between each other via small diffusible chemical signals termed autoinducers. *Pseudomonas aeruginosa* is an opportunistic pathogen involved, among others, in cystic fibrosis complications. Virulence of *P. aeruginosa* relies on its ability to produce a number of autoinducers, including 4-hydroxy-2-alkylquinolines (HAQ). In a cell density-dependent manner, accumulated signals induce the expression of multiple targets, especially virulence factors. This phenomenon, called quorum sensing, promotes bacterial capacity to cause disease. Furthermore, *P. aeruginosa* possesses many multidrug efflux pumps conferring adaptive resistance to antibiotics. Activity of some of these efflux pumps also influences quorum sensing. The present study demonstrates that the MexEF-OprN efflux pump modulates quorum sensing through secretion of a signalling molecule belonging to the HAQ family. Moreover, activation of MexEF-OprN reduces virulence factor expression and swarming motility. Since MexEF-OprN can be activated in infected hosts even in the absence of antibiotic selective pressure, it could promote establishment of chronic infections in the lungs of people suffering from cystic fibrosis, thus diminishing the immune response to virulence factors. Therapeutic drugs that affect multidrug efflux pumps and HAQ-mediated quorum sensing would be valuable tools to shut down bacterial virulence.

## Introduction

Chronic infections caused by the opportunistic pathogen *Pseudomonas aeruginosa* are strongly associated with cystic fibrosis (CF)-related complications such as lung damage and airway obstruction [1]. These complications result from the exacerbated inflammation associated with the chronic infection of cystic fibrotic lungs [2]. Antibiotic therapies aiming at long-term remission are usually inefficient and, consequently, lungs of most individuals suffering from CF are permanently colonized by *P. aeruginosa* [3]. *P. aeruginosa* is resistant to a wide diversity of important antimicrobial agents [4], which is largely due to the activity of multiple multidrug efflux pumps. Among the various types of efflux pumps found in Gram-negative bacteria, the RND (Resistance-Nodulation-cell Division) family plays an important role in the adaptive resistance to antibiotics [5], [6]. In *P. aeruginosa*, activation of the MexEF-OprN RND-type efflux pump gives rise to, among others, chloramphenicol, fluoroquinolones, trimethoprim and triclosan resistance [7]. This efflux pump is encoded by the *mexEF-oprN* operon and is positively controlled by MexT, a transcriptional regulator belonging to the LysR family [8], [9]. The MexS protein also influences the MexEF-oprN efflux state of activity by a mechanism yet to be determined. MexS presents homologies with Zn^2+^-dependent oxidoreductases/deshydrogenases, some of which associated with amino acid metabolism. Mutations in the *mexS* gene lead to a dramatic increase in *mexEF-oprN* transcription [10], [11]. Consequently, some authors have suggested that *mexS*, together with *mexEF-oprN*, could be involved in detoxification [12]. In addition to its role in resistance to antibiotics, the MexEF-OprN efflux pump affects many quorum sensing (QS)-dependent virulence phenotypes (described below).

*P. aeruginosa* QS depends on the transcriptional regulators LasR, RhlR and MvfR (PqsR) as well as on their cognate autoinducer synthases LasI, RhlI and PqsA-D/PqsH, respectively [13]. LasI and RhlI synthesize the autoinducers 3-oxo-dodecanoyl-homoserine lactone (3-oxo-C_12_-HSL) and butanoyl-homoserine lactone (C_4_-HSL) respectively, whereas PqsH catalyzes the final step in the synthesis of 3,4-dihydroxy-2-heptylquinoline [14], [15], [16], known as the *Pseudomonas* Quinolone Signal (PQS) [17]. As the bacterial cell population grows, autoinducers are synthesized, freely diffuse out or are actively exported across Gram negative bacterial membranes and, eventually, exceed a threshold concentration at which point quorum is reached [18], [19]. An autoinducer bind to, and activate, its cognate response regulator which then initiate or repress the transcription of target genes. Activated LasR and RhlR QS regulators exert positive feedback on their own QS system but also they can influence activation of other systems. For instance, LasR stimulates the transcription of the RhlRI and MvfR QS systems, and directly controls the expression of *pqsH* [15], [20], [21]. In this regard, the MvfR system is unique compared to other known QS systems since the expression of its main autoinducer PQS is not directly controlled by its cognate regulator. Instead, PQS concentration relies on 4-hydroxy-2-heptylquinoline (HHQ) production; the product of *pqsABCDE* operon and the direct precursor of PQS [14]. The *pqsABCDE* operon is regulated by MvfR and is involved in the biosynthesis of many other 4-hydroxy-2-alkylquinolines (HAQs) [14], [22].

Interestingly, PQS is poorly synthesized in mutants constitutively expressing *mexEF-oprN*. It was proposed that this signalling molecule, or a precursor, represents a MexEF-OprN substrate [5], [23], [24]. *nfxC* mutants of *P. aeruginosa*, constitutively expressing *mexEF-oprN*, are readily obtained on media containing a high chloramphenicol concentration [7], [25], [26], [27]. QS-related phenotypes characterizing *nfxC* mutants can arise from mutations in the *mexS* gene. In addition to PQS modulation, overexpression of *mexT* and *mexEF-oprN* results in deficiencies in C_4_-HSL, hydrogen cyanide, elastase, pyocyanin and rhamnolipids production, as well as biofilm formation [7], [24], [26], [28]. The reason for these defects is still unknown. It is noteworthy that the 3-oxo-C_12_-HSL signalling molecule is exported by the MexAB-OprM RND-type efflux pump [29], [30].

Thus, to elucidate the molecular phenomena driving QS modulation under MexEF-OprN activity, we used a *mexS* ***^−^*** mutant and various spontaneous *nfxC*-type mutants of *P. aeruginosa* strain PA14. These mutants can overexpress *mexEF-oprN* more than 300-fold compared to the wild-type strain. This strong efflux activity allowed us to demonstrate that an RND-type efflux pump exports the HAQ QS signalling molecule HHQ, the precursor of the PQS, and that this leads to the QS defects characterizing *nfxC*-type mutants.

## Results

### A *P. aeruginosa mexS^−^* mutant overexpressing MexEF-OprN fails to convert most HHQ into PQS

**Figure 1:**
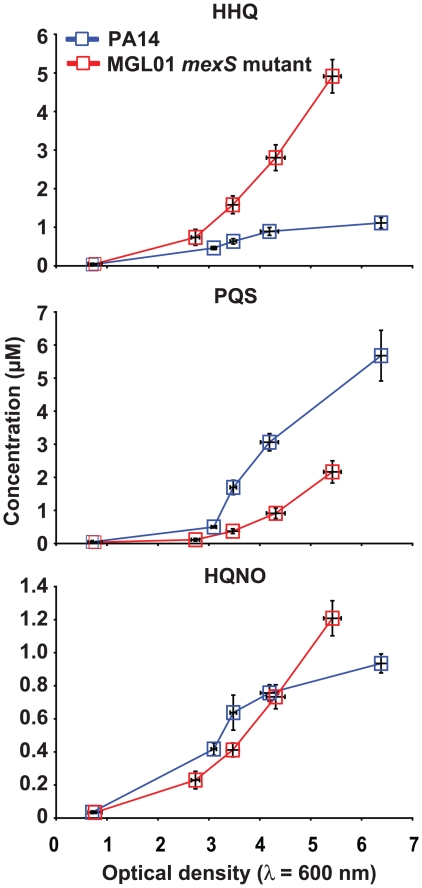
Imbalance of HHQ and PQS production in a PA14 *mexS^−^* mutant (MGL01). HHQ is the direct precursor of PQS but not of HQNO. Compared to the wild-type strain PA14, HHQ accumulates in MGL01 while PQS is poorly produced. HQNO concentrations are not affected by the *mexS* mutation. HAQs were concentrated from ethyl acetate extractions and were quantified by LC-MS/MS. Experiment was achieved using biological triplicates.

To further characterize the effects of MexEF-OprN efflux pump activation on QS, the concentrations of the best known MvfR-dependent QS signaling molecules were compared between the wild-type *P. aeruginosa* strain PA14 and its isogenic *mexS* ***^−^*** mutant (MGL01), which represents an *nfxC* phenotype. Loss of *mexS* results in uncontrolled overexpression of the MexEF-OprN pump (see Experimental procedures) [10], [11]. In MGL01 cultures, the concentration of HHQ, the direct precursor of PQS, is about four-fold higher than in PA14 cultures at an OD_600_ = 5 (Fig. 1). However, and unexpectedly, PQS concentration, at the same growth stage, is about two-fold lower in cultures of MGL01 compared to that of the wild-type strain (Fig. 1). Similarly to HHQ and PQS, the synthesis of the other main HAQ, HQNO, depends on PqsABCD [14], [22]. However, in contrast with PQS, HQNO synthesis is not dependent on the presence of HHQ [14]. Accordingly, there is no significant difference between HQNO concentrations quantified in the wild-type and MGL01 cultures (Fig. 1).

To verify whether perturbed HHQ and PQS production in MGL01 is due to the *mexS* mutation *per se*, a complemented *mexS* ***^−^*** mutant strain MGL01::pML01 was used. As shown in figure S1, HAQ phenotypes are restored to those of the wild-type in the complemented strain. Noteworthy, many *P. aeruginosa* strains carry an 8 bp insertion within the *mexT* gene that causes a frame shift and silences *mexEF-oprN* [10]. Blast analysis against PAO1 *mexT* reveals that strain PA14 possesses a functional *mexT* gene due to the deletion of this insertion, restoring the reading frame. This is in agreement with observations made by Maseda *et al.* (2000) using various PAO1 strains [10]. Nevertheless, in PA14, the MexEF-OprN efflux pump is only expressed at a basal level under experimental conditions (data not shown). Similarly, a *mexE* ***^−^*** mutant (MGL04) displays no defect in HAQ production when compared to wild-type PA14, testifying that basal levels of HAQ efflux *via* MexEF-OprN are not sufficient to affect HAQ phenotypes in the wild-type background (Fig. S1). To determine if HAQ modulation is specific to overexpression of the MexEF-OprN RND-type efflux pump, we verified the production of HAQs in four other constitutively expressing RND efflux pump mutants, *i.e.* in *nfxB* ***^−^***, *mexZ* ***^−^***, *nalC* ***^−^*** and *mexL* ***^−^*** mutants. These mutants overproduce MexCD-OprJ, MexXY-OprM, MexAB-OprM and MexJK-OprM, respectively. We found that none of these other efflux pumps influences HHQ and PQS production (Fig. S2). Together, these results indicate that HHQ and PQS are specifically modulated by MexEF-OprN in the MGL01 *mexS* ***^−^*** mutant.

### Expression of the *pqsH* gene is upregulated in a PA14 *mexS^−^* mutant

**Figure 2:**
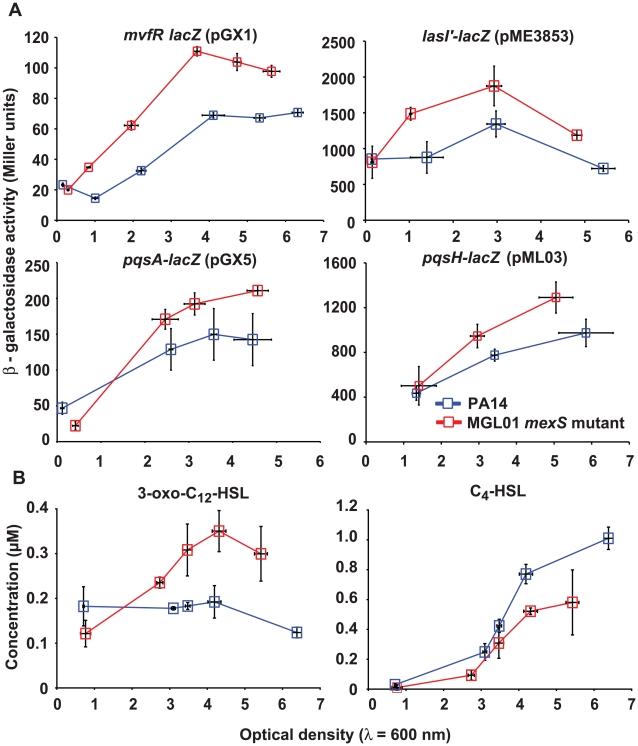
LasRI and MvfR quorum sensing are up-regulated in a PA14 *mexS^−^* mutant (MGL01). (A) Shown is the β-galactosidase activity (Miller units) of transcriptional and translational fusions as a function of cell growth (OD_600_). Notably, transcription of the PQS biosynthetic gene (*pqsH*) is upregulated in MGL01. Experiment was achieved using biological triplicates. (B) Shown are the concentrations of 3-oxo-dodecanoyl-homoserine lactone (3-oxo-C_12_-HSL) and butanoyl-homoserine lactone (C_4_-HSL) as a function of cell growth (OD_600_). AHLs were concentrated from ethyl acetate extractions and were quantified by LC-MS/MS. Experiment was achieved using biological triplicates.

To understand the reason for the HHQ and PQS imbalance characterizing the *mexS* ***^−^*** mutant, the expression patterns of appropriate QS regulators and their related genes were studied using transcriptional and translational reporter fusions. We found that the transcriptional activity of the *mvfR* promoter is nearly two-times higher in MGL01 than in the wild-type strain (Fig. 2A). As expected, the transcription of the *pqsABCDE* operon, which is activated by MvfR, was found to be upregulated as well (Fig. 2A). The molecular basis explaining this increased expression is unknown (discussed below). Since *mvfR* and *pqsH* are positively controlled by the LasRI QS system, it was interesting to investigate the expression of the *lasR* and *lasI* genes in MGL01. The *lasI* translational reporter fusion revealed that the 3-oxo-C_12_-HSL synthase is half-fold more expressed in MGL01 than in PA14 (Fig. 2A). Accordingly, 3-oxo-C_12_-HSL concentrations quantified by LC-MS/MS are higher in MGL01 cultures when compared to PA14 (Fig. 2B). In contrast, C_4_-HSL concentrations are lower in MGL01 cultures (Fig. 2B).

Intriguingly, although PQS, the product of PqsH activity, is produced in lower concentrations in MGL01, *mvfR* and *pqsABCDE* are up-regulated in this strain. Thus, it was interesting to verify whether the decrease in PQS is due to a decreased transcription of *pqsH* in MGL01 strain. This was verified using a *pqsH* promoter-*lacZ* transcriptional fusion chromosomally integrated into the wild-type and MGL01 strains, yielding PA14 (pML03) and MGL01 (pML03) strains, respectively. Unexpectedly, *pqsH* expression is upregulated in MGL01 compared to the wild-type (Fig. 2A). Yet, this was not reflected in a higher production of PQS, although *mexS* ***^−^*** mutant produces high concentrations of the PQS precursor, HHQ (Fig. 1).

### Trans-expression of *pqsH* and HHQ supplementation do not restore PQS production in *mexS^−^* mutants

**Figure 3:**
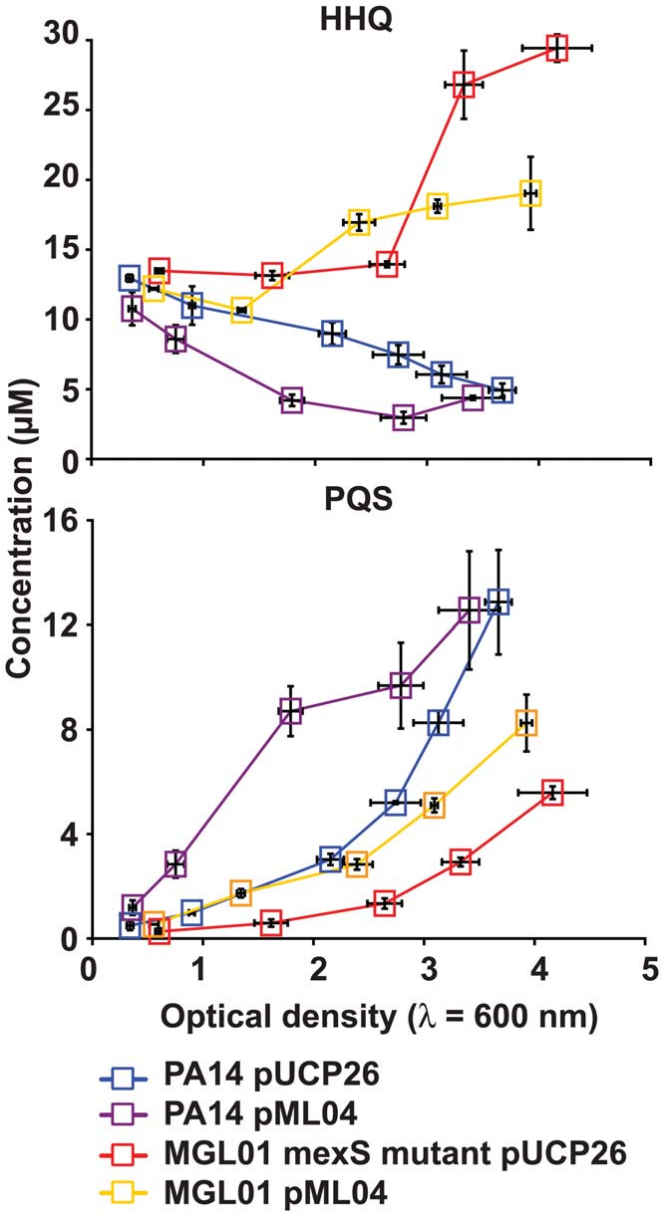
Constitutively expressed *pqsH* (pML04) fails to restore the production of PQS in a PA14 *mexS^−^* mutant (MGL01). Shown are the HAQ concentrations as a function of cell growth (OD_600_). This experiment was conducted in the presence of exogenously added HHQ (41 µM). HAQs were quantified by LC-MS/MS and the experiment was achieved using biological triplicates.

In order to verify whether the reduced PQS production in the *mexS* ***^−^*** mutant is caused by a posttranscriptional mechanism, we introduced a medium copy number plasmid carrying *pqsH* under a constitutive promoter (pML04) into the wild-type and MGL01 strains, giving strains PA14 (pML04) and MGL01 (pML04) respectively. Also, to promote HHQ availability to PqsH, exogenous HHQ was added to the cultures. As expected, PA14 (pML04) rapidly consumes exogenous HHQ to produce PQS while the PA14 (pUCP26) control strain presents a more gradual HHQ consumption with a corresponding simultaneous PQS production (Fig. 3). In contrast, we observed a slight decrease in available HHQ in MGL01 (pML04), with a concomitant limited conversion into PQS (Fig. 3). Thus PQS production is only partially restored in a *mexS* ***^−^*** background by *pqsH* gene upregulation and exogenous supplementation of HHQ.

### Constitutively expressing MexEF-OprN mutant cells contain less HHQ than wild-type cells

**Figure 4:**
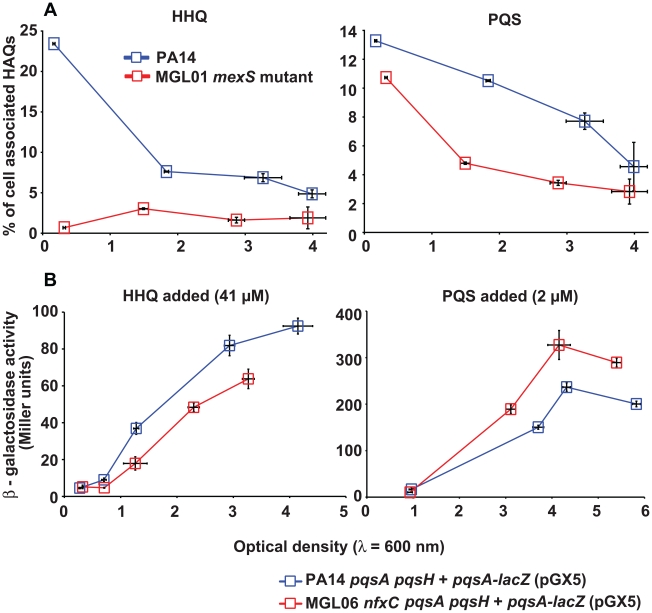
HHQ is exported out of constitutively expressing MexEF-OprN efflux cells (MGL01). (**A**) Shown is the percentage of cell-associated HHQ or PQS as a function of cell growth (OD_600_). Here, we show that MGL01 cells contain less cell-associated HHQ and PQS when compared to the PA14 wild-type strain. This is in contrast with the observation that HHQ concentrations are higher in MGL01 than in PA14 cultures (Fig. 1). HAQs were quantified by LC-MS/MS and experiment was achieved using biological triplicates. (B) Shown is the β-galactosidase activity (Miller units) of a *pqsA-lacZ* transcriptional reporter as a function of cell growth (OD_600_). This experiment was conducted in the presence of exogenously added HHQ (41 µM) or PQS (2 µM). Experiment was achieved using biological triplicates.

The above results demonstrate that HAQ concentrations in MGL01 are not only directly influenced by the transcriptional and translational status of *lasRI*, *mvfR*, *pqsABCDE* and *pqsH* genes but also by another unidentified mechanism. We thus hypothesized that the activity of the MexEF-OprN efflux pump directly modulates HAQ intracellular concentrations, the fact that could explain the weak PQS synthesis by the *mexS* ***^−^*** mutant cells. This was first verified by directly quantifying cell-associated HHQ and PQS molecules in PA14 and MGL01. As shown in figure 4A, proportions of cell-associated HHQ are lower in MGL01 than in the wild-type strain PA14. This result is even more striking when considering that MGL01 is a hyper HHQ producer (Fig. 1). Nonetheless, a higher concentration of cell-associated PQS is observed in the wild-type strain PA14 (Figure 4A), which is consistent with the higher production of PQS in PA14 cultures (Fig. 1).

In light of these results, we compared the intracellular availability of PQS and HHQ for MvfR binding under MexEF-OprN overexpression. PQS or HHQ was exogenously added to cultures of an HAQ-deficient PA14 strain (*pqsA* ***^−^*** *pqsH* ***^−^*** double mutant), or of its isogenic *nfxC* mutant (MGL02), carrying the *pqsA-lacZ* transcriptional reporter plasmid pGX5 [31], [32]. We found that addition of PQS results in a stronger induction from the *pqsA* promoter in MGL02 (pGX5) than in PA14 *pqsA* ***^−^*** *pqsH* ***^−^*** (pGX5) (Fig. 4B). This observation is likely to result from the strong activating effect of PQS on the MvfR transcriptional regulator in this PA14 *nfxC* mutant [14], [32]. Indeed, as described previously, the MGL01 *mexS* ***^−^*** mutant is characterized by a stronger induction from the *pqsA* promoter than the wild-type strain (Fig. 2). Strickingly, MGL02 (pGX5) presents a lower HHQ-dependent induction of the *lacZ* reporter gene, when compared to that of the PA14 *pqsA* ***^−^*** *pqsH* ***^−^*** double mutant (Fig. 4B). These results indicate that HHQ is less efficient in activating MvfR when MexEF-OprN is constitutively overexpressed, suggesting that HHQ is rapidly pumped out of the cells, reducing its availability for MvfR activation.

### The MexEF-OprN efflux pump exports HHQ

**Figure 5:**
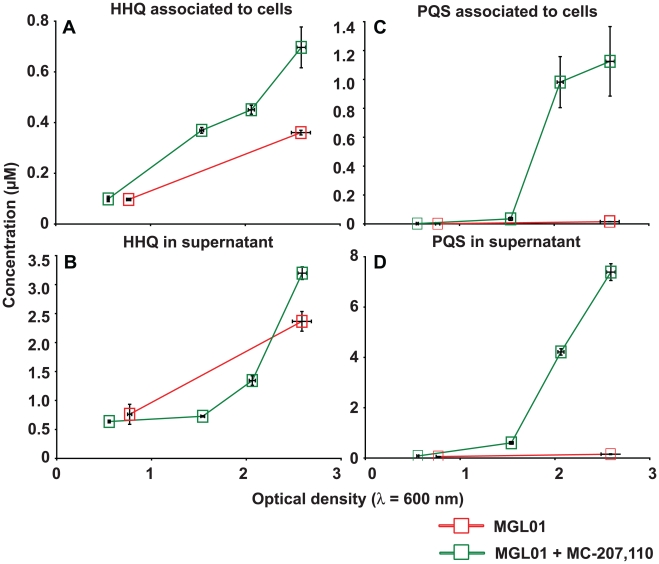
Rapid export of HHQ by the MexEF-OprN efflux pump reduces its availability to PqsH and consequently, PQS biosynthesis. The efflux pump inhibitor (EPI) MC-207,110 causes HHQ concentrations to (A) increase within PA14 *mexS^−^* mutant (MGL01) cells and to (B) decrease in supernatants. Accordingly, (D and C) the EPI promotes PQS production in MGL01. The EPI was initially added to synchronized MGL01 cultures (OD_600_ = 0.05) at a concentration of 20 µg/ml. Another 20 µg/ml was added after two hours of growth. HAQs were quantified by LC-MS/MS and experiment was achieved using biological triplicates.

To further verify this hypothesis, we quantified HAQ concentrations while blocking the efflux activity of MexEF-OprN. This was accomplished by assaying HAQs in cultures exposed to an efflux pump inhibitor (EPI): the MC-207,110 molecule [33]. Because of its growth-inhibitory effect, MC-207,110 was used at low concentrations (see experimental procedures). HAQs from supernatant as well as from washed cells were quantified by LC-MS/MS. Remarkably, cell-associated HHQ increased in EPI-treated MGL01 cultures (Fig. 5A), whereas the corresponding culture supernatants showed lower HHQ concentrations when compared to untreated cultures (Fig. 5B). Moreover, PQS concentrations are considerably higher in both of the washed cell samples and their corresponding supernatants in EPI-treated MGL01 cultures when compared to controls (Figure 5C–D). Taken together, these results support our hypothesis that, in *mexS* ***^−^*** mutants, where the MexEF-OprN efflux pump is constitutively overexpressed, much less intracellular HHQ is available to the PqsH enzyme for PQS synthesis.

**Figure 6:**
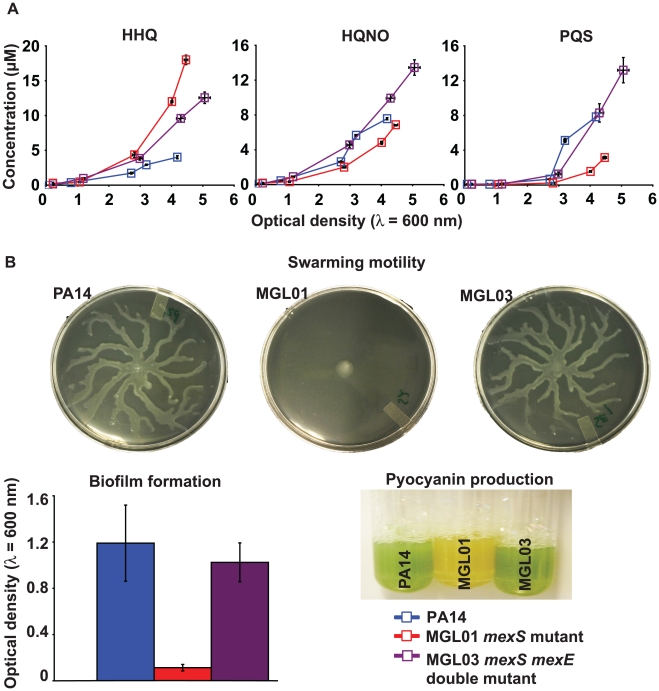
The phenotypes of a *mexS^−^* mutant are directly explained by the overexpression of the MexEF-OprN efflux pump. In a PA14 *mexS* ***^−^*** mutant background, a mutation in *mexE* restores (A) HAQ concentrations as well as QS-related virulence phenotypes (B) to that of the wild-type. HAQs were quantified by LC-MS/MS and experiments were achieved using biological triplicates.

Finally, to conclusively confirm the importance of the MexEF-OprN efflux activity in the modulation of HAQ production, we constructed a *mexS* ***^−^*** *mexE* ***^−^*** double mutant of strain PA14, giving strain MGL03. As expected, figure 6 is showing that PQS production is increased to wild-type levels by the inactivation of *mexE* while HHQ concentrations are decreased, although not fully restored to that of the wild-type, in MGL03 (discussed below). Moreover, the *mexE* knockout restores QS-related virulence phenotypes in MGL01 such as swarming motility, pyocyanin production and biofilm formation (Fig. 6). As expected *mexS* ***^−^*** complementation in MGL01 with pML01 also restores the phenotypes to that of the wild-type (Fig. S3). Taken together, these results confirm HHQ as a substrate of the MexEF-OprN efflux system, which affects its intracellular availability for PqsH-mediated conversion into PQS and consequently, modulate QS-dependent virulence traits.

## Discussion

*P. aeruginosa* is highly resistant to a large spectrum of antimicrobial agents as it possesses, among other factors, an array of both constitutively active and inducible efflux pumps. In addition to their well-studied role as major antibiotic resistance determinants, these pumps are currently thought to play other unexpected functions, including QS modulation [29], [30], [34], [35], [36]. The role of the MexEF-OprN efflux pump in the *P. aeruginosa* biology is closely related to the *mexS* gene, whose function is still misunderstood. K. Poole (2005) has proposed that MexS could serve as a detoxifying enzyme for a yet unidentified metabolite [12]. Additionally, the MexEF-OprN efflux pump could be important for this metabolite secretion in non functional *mexS* cells [12]. However, the natural substrate of MexEF-OprN is unknown. Based on our results and hypothesis put forward by K. Poole, it is tempting to speculate that such a molecule could be structurally related to quinolones. Indeed, the present study shows that the MexEF-OprN efflux pump exports the PQS precursor, HHQ.

Results presented in figure 1 illustrate well the link that exists between HHQ synthesis and PQS accumulation. In the wild-type strain PA14, PQS accumulates while the availability of HHQ remains stable and low, indicating that the synthesis rate of HHQ is similar to that of PQS, as we have previously reported [14]. This equilibrium is disturbed in the *mexS* ***^−^*** mutant, MGL01, since HHQ accumulates while PQS is poorly synthesized (Fig. 1). We observed that the LasR-controlled QS genes, including *mvfR*, are upregulated in MGL01 (Fig. 2A). However, *mvfR* upregulation alone cannot explain why the *pqsABCDE* operon, involved in HHQ synthesis, is upregulated in the weak PQS producer strain MGL01. This is intriguing, especially when considering that PQS is the primary activating ligand of MvfR. Unknown factors, independent of PQS or HHQ, in *mexS* ***^−^*** mutants might be exerting a positive effect on the transcription of *pqsABCDE*. This hypothesis is currently under investigation in our laboratory. Still, it is plausible that upregulation of LasR-controlled QS genes (*lasR* regulon) might result from the increased 3-oxo-C_12_-HSL concentration observed in MGL01 cultures, as shown in figure 2B [30], [37]. Interestingly, MexT, which is highly expressed in *nfxC*-type mutants, blocks the RhlR/C_4_-HSL-dependent activation of the *mexAB-OprM* operon, an efflux pump involved in 3-oxo-C_12_-HSL export [29], [30], [37]. Therefore, MexT-dependent downregulation of *mexAB*-OprM could well be responsible for the 3-oxo-C_12_-HSL accumulation seen with MGL01 cells, thus stimulating, in a positive feedback loop fashion, the LasR-controlled genes. Being a part of the LasR regulon, *pqsH* expression is upregulated in the MGL01 *mexS* ***^−^*** mutant, yet, the overproduced HHQ is poorly converted into PQS. Even higher expression of the *pqsH* gene from a constitutive p*lac* promoter fails to complement the MGL01 PQS production defect (Fig. 3). Together, these results strongly suggest that most HHQ produced by MGL01 should be rapidly secreted out of cells and kept in the extracellular milieu (Figs. 1 and 3). Accordingly, in a *nfx*C background, exogenously added HHQ fails to activate MvfR and consequently, to induce the *pqsA-lacZ* reporter construct (Fig. 4b).

At this stage of the study, results and clues we had pinpointed toward a role for the MexEF-OprN efflux pump in the export of HHQ. We thus designed experiments to confirm this hypothesis. We first showed that *mexS* ***^−^*** mutant cells, which produce much more HHQ than the wild-type, contain less cell-associated HHQ than the wild-type cells. HAQs are molecules that are thought to diffuse across, and get trapped into, lipid membranes. For instance most PQS is cell-bound [38]. PQS was also shown to bind lipid A molecules, while HHQ does not [39]. Thus, in MGL01, putative lipid A modifications are unlikely be responsible for the observed low cell-bound HHQ concentration. Instead, it is very probable that MexEF-OprN constitutive activity, which characterizes *mexS* ***^−^*** mutant cells, causes a rapid export of HHQ away from the intracellular PqsH enzyme, explaining why PQS is poorly synthesized in MGL01. Accordingly, the efflux pump inhibitor (EPI) MC-207,110 increases HHQ accumulation in MGL01 cells, seemingly just enough to induce PQS synthesis which must be concomitant with HHQ consumption (Fig. 5A) [33]. Finally, we show that knocking out *mexE* gene in the MGL01 background (MGL03 strain) restores PQS production to that of the wild-type levels, as well as some QS-dependent virulence phenotypes such as swarming motility, pyocyanin production and biofilm formation (Fig. 6B) [11], [24], [26], [30], [40]. Interestingly, although PQS synthesis is restored in MGL03 strain, there is an upregulation in the expression of *pqsABCDE* demonstrated by the accumulation of HHQ when compared to the wild-type (Fig. 6A). This observation strengthens our hypothesis that, in *mexS* ***^−^*** mutants, the *pqsABCDE* operon may be subject to genetic control other than that exerted by the MvfR-PQS complex. Furthermore, results obtained with strain MGL03 are in accordance with those of Favre-Bonté *et al.* (2003), who showed that a *mexE* knock-out restores the biofilm as well as C_4_-HSL deficiencies characterizing the *nfxC* strain PAO1-BI relative to the wild-type phenotypes [23]. Similarly, Cosson *et al.* (2002) reported that such double mutant is as virulent as the wild-type *P. aeruginosa* in infection models [41]. Together, our results validate the role of the MexEF-OprN apparatus *per se* in the export of HHQ. Our finding finally validates the hypothesis put forward by Köhler *et al.* in 2001 that this efflux pump exports a precursor of PQS [24].

The MexEF-OprN is apparently inactive in most *P. aeruginosa* strains, permitting expression of QS-dependent virulence factors [10]. Nevertheless, isolates from chronic CF infections were shown to carry multiple mutations that simultaneously silence expression of QS-related virulence factors (*e.g. lasR* ***^−^*** and *pqsABCDE* ***^−^*** mutants) and enhance resistance to antibiotics [27]. Likewise, some studies noted the increased expression of *mexEF-oprN* in clinical isolates of *P. aeruginosa*, suggesting that a positive pressure occurs *in vivo* for *nfxC* clones selection [25], [42], [43], [44]. Moreover, HHQ was found to be more abundant than PQS in a mammalian infection model, which is reminiscent of MGL01 *mexS* ***^−^*** mutant HAQs phenotypes [32], [45]. Since QS is essential for *P. aeruginosa* virulence in various infection models, we believe that the expression status of the MexEF-OprN efflux pump could be an important determinant permitting the switch from an acute to a chronic infection “mode”. Obviously, infected hosts can be submited to a strong antibiotherapy that selects for multiresistant *P. aeruginosa*. Interestingly however, *mexEF-oprN* constitutive expression has also been reported in the absence of selective pressure. Transcriptomic data from *P. aeruginosa* interacting with primary normal human airways epithelial cells revealed overexpression of the *mexEF-oprN* operon [46]. Also, frequency of *nfxC* emergence was seen to be 10-fold higher *in vivo* (model of acute pneumonia in rat), without antibiotics pressure, than in conventional culture media [43]. In light of these studies, we believe that prolonged *P. aeruginosa* exposure to some *in vivo* conditions alone might suffice to induce *mexEF-oprN* expression or select for *nfxC* mutants. Likewise, Fetar *et al.* (2010) recently showed that *mexEF-OprN* transcription is induced by *in vitro* nitrosative stress, a condition also known to occur during pulmonary infections [47]. Thus, in addition to some unknown environmental cues, selective pressures from *in vivo* environments might promote *mexEF-oprN* expression and chromosomal stability. In conclusion, we propose that the MexEF-OprN efflux pump constitutes an important machinery that modulates the virulence of *P. aeruginosa* through the export of specific QS regulatory molecules, especially HHQ.

## Methods

### Experimental procedures

#### Bacterial strains, plasmids and media

Strains and plasmids used in this study are listed in Table 1. Bacteria were grown in Tryptic Soy Broth (TSB) (Difco) at 37°C. *nfxC* mutants were selected on TSB agar plates containing 500 µg/ml chloramphenicol. HHQ was synthesized as described [38] and was added to cultures at a concentration of 41 µM, when indicated. When required, antibiotics were used at the following final concentrations: carbenicillin, 50 µg/ml; chloramphenicol, 500 µg/ml; gentamycin, 50 µg/ml; kanamycin, 50 µg/ml; tetracycline, 75 µg/ml in liquid TSB and 125 µg/ml in TSB agar. All antibiotics were from Sigma except for carbenicillin (Duchefa).

**Table 1:** Bacterial strains and plasmids.

Bacterial strains/Lab. No	Relevant characteristics^a^	Reference or source
*Escherichia coli* strain		
SM10λ*pir*	*thi-1*, *leu*, *tonA*, *lacY*, *supE*, *recA*::*RP4-2-Tc*::*Mu*, λ*pir*, Km^r^	[54]
*Pseudomonas aeruginosa* strains		
PA14	Clinical isolate UCBPP-PA14	[61]
PA14 *mexS*::Tn*5*/ED1188	*mexS::*IS*lacZ*/hah, Tet^r^	This study
MGL01/ED1189	PA14 *mexS* Leucine185::63 (from ED1188)	This study
PA14 *pqsA* ***^−^*** * pqsH* ***^−^***	*pqsA*::Tn*phoA*, Km^r^; *pqsH*::*aacC*1, Gm^r^	[32]
MGL02/ED1194	*nfxC* mutant derived from PA14 *pqsA^−^pqsH^−^*	This study
MGL03/ED1195	ED1188+Δ*mexE*, Tet^r^	This study
MGL04/ED1395	Δ*mexE*	This study
PA14 *mexL^−^*	MexJK-OprM constitutive strain	[62]
PA14 *mexZ^−^*	MexXY-OprM constitutive strain	[62]
PA14 *nalC^−^*	MexAB-OprM constitutive strain	[62]
PA14 *nfxB^−^*	MexCD-OprJ constitutive strain	[62]
Plasmids		
mini-CTX1	Tet^R^, *ori*, *int*, *oriT*, Ω-FRT-*attP*-MCS	[52]
mini-CTX-*lacZ*	Tet^R^, *ori*, *int*, *oriT*, Ω-FRT-*attP*-MCS	[55]
pCRE2	pUT derivative harboring the phage P1 *cre* gene	[49]
pEX18Ap	Ap^r^; oriT+ sacB+, gene replacement vector with MCS from pUC18	[53]
pFLP2	Source of FLP recombinase, *sacB*, *oriT*, *rep*, Ap^r^	[53]
pGEM-T easy	Cloning vector	Promega
pGX1	*mvfR–lacZ* transcriptional fusion, Cb^r^	[58]
pGX5	*pqsA-lacZ* transcriptional fusion, Cb^r^	[31]
pIT2	IS*lacZ*/hah, Tet^r^	[48]
pME3853	*lasI-lacZ* translational fusion, Tet^r^	[63]
pML01	mini-CTX1::*mexS*	This study
pML02	pEX18Ap::Δ*mexE*	This study
pML03	*pqsH* promoter in mini-CTX-*lacZ*	This study
pML04	pUCP26::*pqsH*	This study
pUCP26	Tet^R^, *ori*, *rep*, p*lac*-MCS-*lacZ*α	[56]

a Ampicillin, Ap^r^; Carbenicillin, Cb^r^; Chloramphenicol, Cm^r^ ; Gentamycin, Gm^r^; Kanamycin, Km^r^; Tetracycline, Tet^r^.

### Construction of plasmids and mutant strains

#### Construction of the *mexS^−^* mutant

The *mexS* ***^−^*** mutant used in this study was obtained from random mutagenesis using the pIT2 plasmid (ISlacZ/hah transposon) [48] during a screening for swarming deficient mutants (J. Tremblay and E. Déziel, unpublished data). This mutant is designated *mexS*::Tn*5*. The tetracycline resistance marker was excised from the insertion site using the Cre recombinase harbored by the pCRE2 plasmid; leaving a 63 amino acids insertion at leucine 185 [49]. This mutant strain is designated MGL01. To confirmed constitutive expression of the *mexEF-oprN* operon in MGL01, posttranscriptional status of *mexE* mRNA was assessed by qRT-PCR. Compared to the wild-type strain PA14, transcription of the *mexE* gene is increased 21- and 12-fold at OD_600_ of 1.4 and 4.0, respectively. The fold change was calculated using the 2^−ΔΔ*C*t^ method [50]. The *nadB* gene was used as a housekeeping control [51]. To complement the *mexS* ***^−^*** mutation, the *mexS* gene was amplified using the inMexT-F (5′-CGACGTCGTCGGAAAGGCCGATG-3′) and PA14_32440-R (5′-CTAGCCAGGTTGCACGATCATCCAAGAC-3′) primers and cloned into the pGEM-T easy vector (Promega, Madison, WI, USA). The NotI fragment of the pGEM-T easy::*mexS* construct was then cloned into the NotI restriction site of the mini-CTX1 plasmid [52], giving pML01. This construct was then introduced into competent *E. coli* SM10λ*pir* for mobilization into *P. aeruginosa* MGL01 by conjugation, to give MGL01 (pML01).

#### Construction of the *mexS^−^ mexE^−^* double mutant

Phusion High fidelity DNA polymerase (New England BioLabs, Inc., Ipswich, MA, USA) was used for all DNA amplifications. A genomic fragment (2488 bp) containing the *mexE* gene was amplified using primers pmexE-F (5′-CGAGGAACTGGAGAAATTCG-3′) and mexE-R2 (5′-ACAACTGGAAGCTGGTATCG-3′) and cloned into the pGEM-T easy vector. An in-frame deletion within the *mexE* gene was performed by PCR using the outward primers mexE5′Ext-kpnI (5′-GGTACCGAATTCGTCCCACTCGTTCAG-3′) and mexE3′Ext-kpnI (5′-GGTACCGATCCGCAGAAGGTCGAGATG-3′), both containing a 5′-end KpnI restriction site. The KpnI digest of the PCR product was circularized using T4 ligase (NEB). Then, the SacI-SphI fragment from the resulting construct was inserted into the suicidal vector pEX18Ap cut with the same enzymes, to generate pML02 [53]. pML02 was mobilized into PA14 *mexS*::Tn*5* using the *E. coli* SM10λ*pir* donor strain [54]. Merodiploids were selected on TSB agar containing carbenicillin and tetracycline and then Δ*mexE* mutants were resolved on plates containing 7% sucrose. The *mexS* ***^−^*** *mexE* ***^−^*** double mutant is designated strain MGL03. The PA14 *mexE* ***^−^*** single mutant (MGL04) was generated using the same strategy.

#### Construction of chromosomal *pqsH-lacZ* transcriptional reporter

The *pqsH* promoter region was amplified using the p*pqsH*-F (5′-GGTACCTAAGGGGTTGACAGGAGC-3′) and p*pqsH*-R (5′- GGATCCCCGTTGCTCCTTAGCAGC-3′) primers, which contain the restriction sites KpnI and BamHI, respectively (underlined). The resulting 512 pb fragment was cloned into the corresponding sites of the mini-CTX-*lacZ* plasmid [55], giving pML03, which was then introduced into competent *E. coli* SM10λ*pir* for mobilization into *P. aeruginosa* PA14 and MGL01 by conjugation, to give PA14 (pML03) and MGL01 (pML03), respectively. Clones carrying chromosomal insertion of the mini-CTX constructions were selected on tetracycline TSB plates. The tetracycline resistance cassette was excised from the chromosome using the flipase expressed from plasmid pFLP2 [53].

#### Construction of constitutively expressed *pqsH* gene

The *pqsH* gene was amplified using primers pqsH-sacI-F (5′-GAGCTCATGACCGTTCTTATCCAGGG-3′) and the pqsH-HindIII-R (5′-AAGCTTCTACTGTGCGGCCATCTCA-3′). The resulting 1149 bp fragment was cloned into the SacI and HindIII restriction sites of the pUCP26 plasmid [56], giving pML04.

#### β-galactosidase assay

β-galactosidase activity was measured as described previously by Miller with slight modifications [57]. Briefly, cells were grown in TSB to various cell densities and then diluted in Z buffer (Na_2_HPO_4_ 0.06 M; NaH_2_PO_4_ 0.04 M; KCl 0.01 M; MgSO_4_ 0.001 M; β-mercaptoethanol 0.05 M; pH 7). Cells were permeabilized by the addition of one drop of 0.1% SDS and two drops of chloroform. Then, 200 µL of ONPG 4 mg/ml was added to each reaction. Color development was monitored at 420 nm and β-galactosidase activity was expressed in Miller units (MU), calculated as follows: 1,000×OD_420_/*T* (min)×*V* (ml)×OD_600_.

#### Detection and measurements of HAQs by LC/MS

Except when specified, 300 µL culture samples were taken at regular intervals, used for determination of growth (OD_600_), and mixed with 300 µl methanol containing 75 µM of tetradeutero-PQS and 40 µM of tetradeutero-HHQ for final concentrations of 37.5 and 20 µM respectively, as internal standards [38]. After centrifugation, samples were directly injected for LC separation on an Agilent HP1100 HPLC system equipped with a 3×150 mm C8 Luna reverse-phase column (Phenomenex). A 1% acidified water/acetonitrile gradient was used as the mobile phase at a flow rate of 0.4 ml⋅min^−1^, split to 10% with a Valco Tee. A Quattro II (Waters) triple-quadrupole MS was used for molecule detection. Data acquisition was performed in positive ion mode with a scanning range of 100–400 Da. Precise quantification of HAQs was performed by MS/MS, as described previously [22], [58].

#### Efflux pump inhibitor assays

The MC-207,110 molecule is an efflux pump inhibitor (EPI) and was kindly provided by Olga Lomovskaya from Mpex Pharmaceuticals [33]. The MexEF-OprN efflux pump is absolutely essential for the high chloramphenicol resistance feature of *nfxC* mutants, which are readily isolated on chloramphenicol plates. We thus evaluated the efficiency of MC-207,110 to decrease the activity of MexEF-OprN by its effect on the emergence of *nfxC* colonies. This was conducted by plating 50 µL of overnight wild-type cultures onto TSB agar plates containing 500 µg/ml of chloramphenicol (a concentration only permissive to *nfxC* mutants), supplemented or not with MC-207,110 (20 µg/ml). Plates containing this low concentration of the EPI showed a 50% reduction in the emergence of *nfxC* colonies after 24 h of incubation at 37°C when compared to control plates (data not shown), confirming the efficiency of this molecule in blocking the activity of MexEF-OprN. Thus, MC-207,110 was initially added to synchronized MGL01 cultures (OD_600_ = 0.05) at a concentration of 20 µg/ml. To minimize growth inhibition effect caused by the EPI, a supplemental 20 µg/ml was added only after 2 h of growth. Twenty µg/ml of MC-207,110 has no effect on bacterial growth whereas 40 µg/ml results in a slight reduction of MGL01 growth (Fig. S4). For LC-MS/MS analyses, whole culture, supernatant and PBS washed cell samples were used.

#### Swarming and biofilm assays

Swarming motility and biofilm assays were performed as described previously [59], [60]. Swarm plates were incubated at 30°C for 16 h. Biofilm assay was conducted at 37°C for 72 h using TSB for bacterial growth.

## Supporting Information

Figure S1 **(A)** ***mexS trans*** **-complementation restores HAQ imbalance occuring in a PA14** ***mexS^−^*** **mutant.** (B) The wild-type HAQs production is not affected by *mexE* mutation. HAQs were quantified by LC-MS/MS and experiment was achieved using biological triplicates.
(TIF)

Figure S2 **Mutants overexpressing other RND-type efflux pumps do not display any defect in HAQ production.** Shown are the HAQ concentrations as a function of cell growth (OD_600_). HAQs were quantified by LC-MS/MS and experiment was achieved using biological triplicates.
(TIF)

Figure S3 ***mexS trans*** **-complementation restores QS-related virulence phenotype defects of a PA14** ***mexS^−^*** **mutant.** Experiments were achieved using biological triplicates.
(TIF)

Figure S4 **Bacterial growth inhibition by the EPI MC-207,110.** Shown is the cell growth (OD_600_) as a function of time, as measured using a Bioscreen C apparatus (Oy Growth Curves Ab Ltd) (A) the *P. aeruginosa* wild-type strain PA14, (B) the MGL01 *mexS* ***^−^*** mutant. Experiments were achieved using biological triplicates.
(TIF)
