# Supplementary figures and images for: MexEF-OprN Efflux Pump Exports the Pseudomonas Quinolone Signal (PQS) Precursor HHQ (4-hydroxy-2-heptylquinoline)
Source: PLoS One. 2011 Sep 21;6(9):e24310. doi: 10.1371/journal.pone.0024310 (PMC3177830; doi:10.1371/journal.pone.0024310)

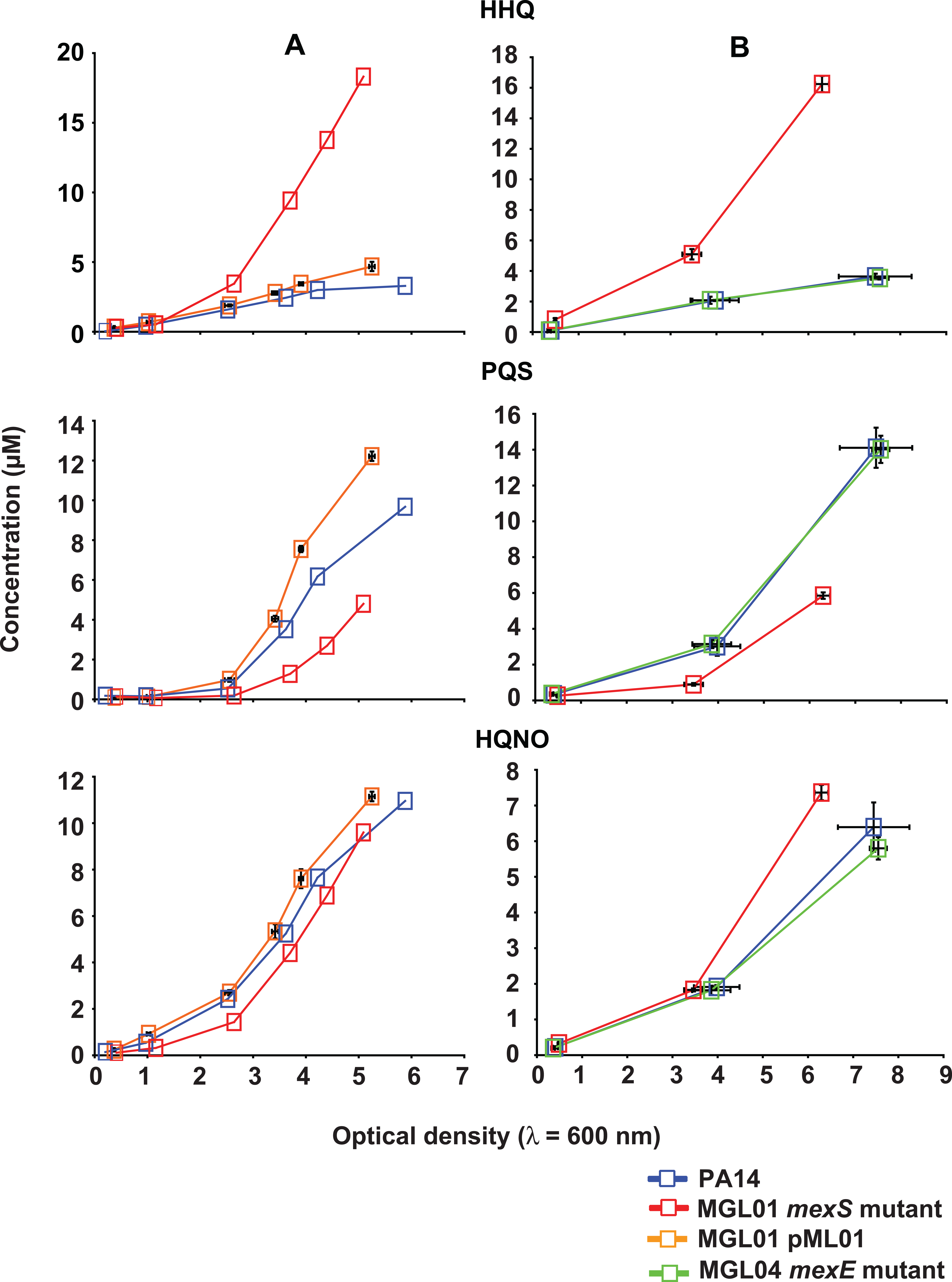

Supplement: Figure S1 — (A) mexS trans -complementation restores HAQ imbalance occuring in a PA14 mexS− mutant. (B) The wild-type HAQs production is not affected by mexE mutation. HAQs were quantified by LC-MS/MS and experiment was achieved using biological triplicates. (TIF) [file pone.0024310.s001.tif]

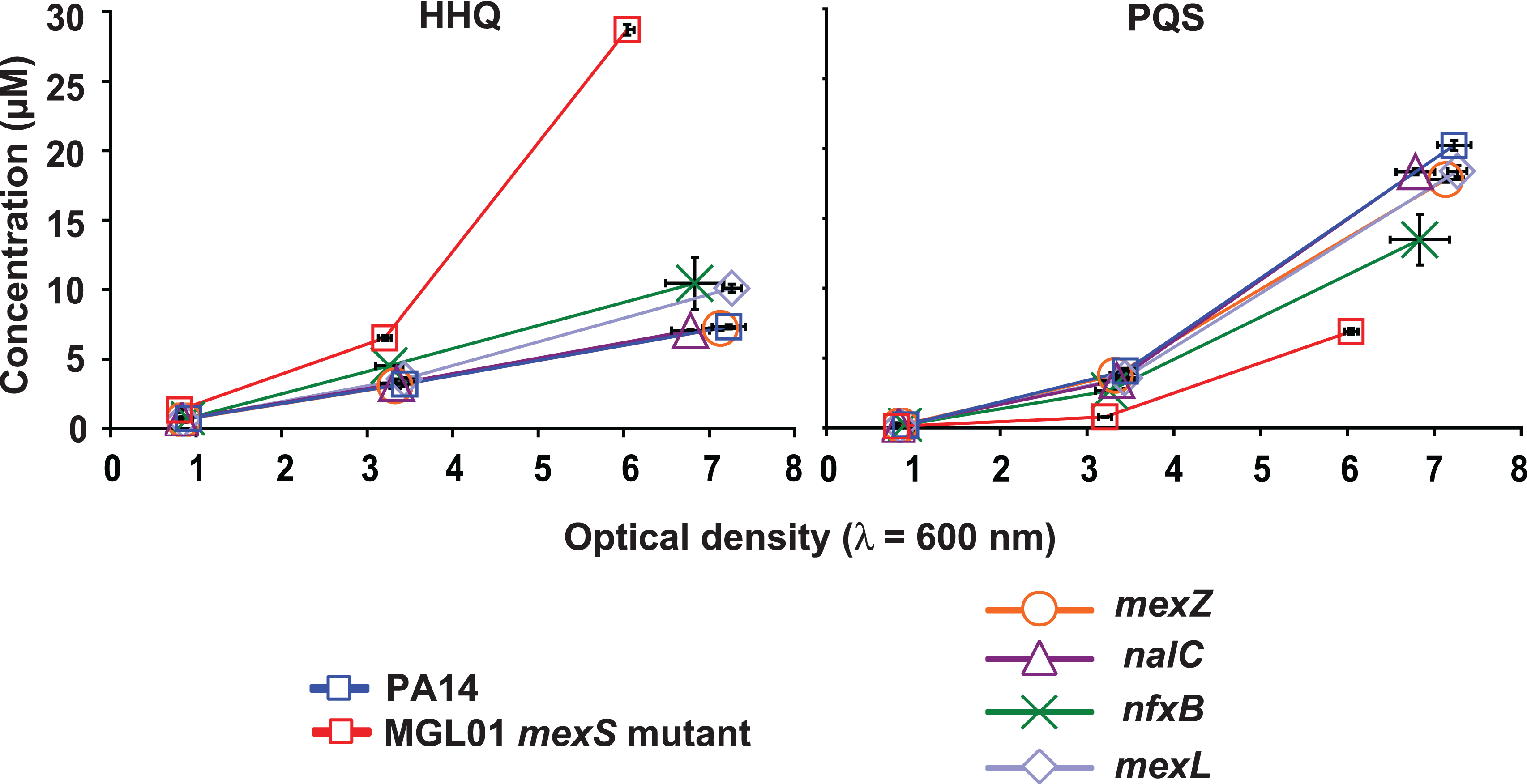

Supplement: Figure S2 — Mutants overexpressing other RND-type efflux pumps do not display any defect in HAQ production. Shown are the HAQ concentrations as a function of cell growth (OD600). HAQs were quantified by LC-MS/MS and experiment was achieved using biological triplicates. (TIF) [file pone.0024310.s002.tif]

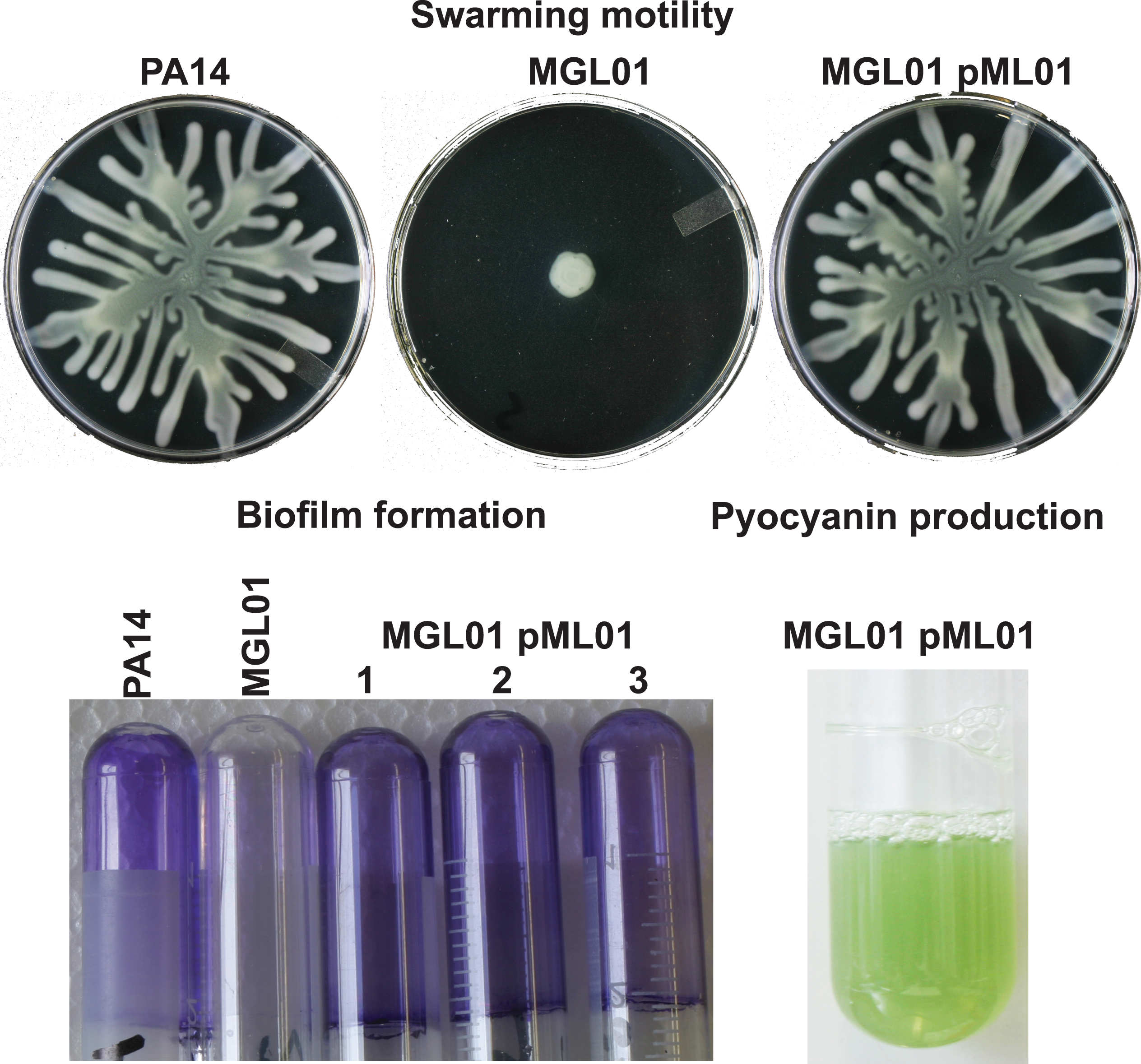

Supplement: Figure S3 — mexS trans -complementation restores QS-related virulence phenotype defects of a PA14 mexS− mutant. Experiments were achieved using biological triplicates. (TIF) [file pone.0024310.s003.tif]

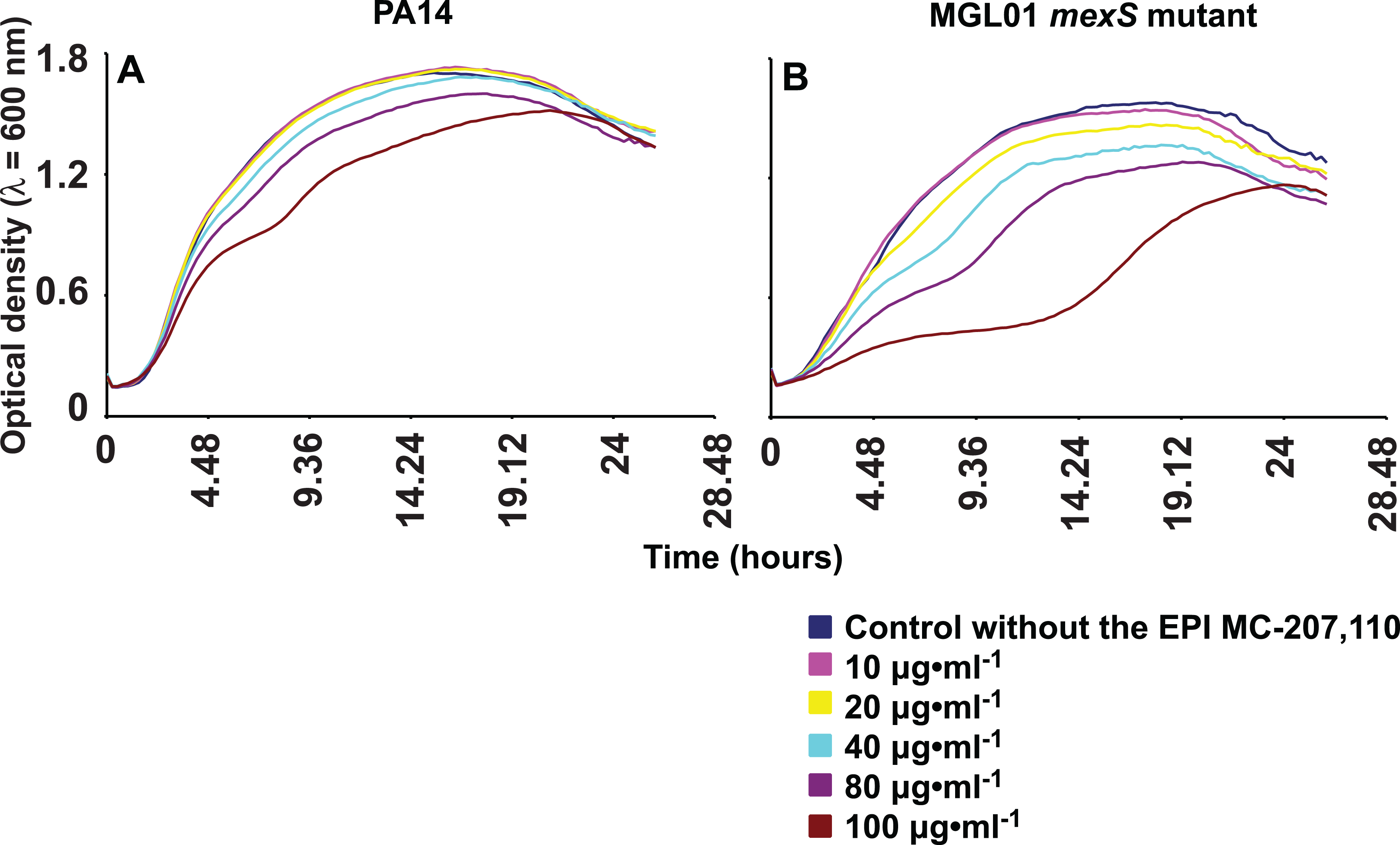

Supplement: Figure S4 — Bacterial growth inhibition by the EPI MC-207,110. Shown is the cell growth (OD600) as a function of time, as measured using a Bioscreen C apparatus (Oy Growth Curves Ab Ltd) (A) the P. aeruginosa wild-type strain PA14, (B) the MGL01 mexS − mutant. Experiments were achieved using biological triplicates. (TIF) [file pone.0024310.s004.tif]
